# Implementation and prospective performance evaluation of an intraoperative duration prediction model using high throughput real-time data

**DOI:** 10.1016/j.bjao.2024.100285

**Published:** 2024-05-07

**Authors:** York Jiao, Thomas Kannampallil

**Affiliations:** 1 Department of Anesthesiology, Washington University School of Medicine, St. Louis, MO, USA; 2 Institute for Informatics, Data Science, and Biostatistics, Washington University School of Medicine, St. Louis, MO, USA

**Keywords:** machine learning, perioperative medicine, statistical models, surgical duration

## Abstract

**Background:**

Accurate real-time prediction of intraoperative duration can contribute to improved perioperative outcomes. We implemented a data pipeline for extraction of real-time data from nascent anaesthesia records and silently deployed a predictive machine learning (ML) algorithm.

**Methods:**

Clinical variables were retrieved from the electronic health record via a third-party clinical decision support platform and contemporaneously ingested into a previously developed ML model. The model was trained using 3 months data, and performance was subsequently evaluated over 10 months using continuous ranked probability score.

**Results:**

The ML model made 6 173 435 predictions on 62 142 procedures. Mean continuous ranked probability score for the ML model was 27.19 (standard error 0.016) min compared with 51.66 (standard error 0.029) min for the bias-corrected scheduled duration. Linear regression did not demonstrate performance drift over the testing period.

**Conclusions:**

We implemented and silently deployed a real-time ML algorithm for predicting surgery duration. Prospective evaluation showed that model performance was preserved over a 10-month testing period.

Accurate prediction of intraoperative duration can contribute to improved operational and clinical outcomes.^1^, ^2^, ^3^, ^4^, ^5^ Many statistical^6^, ^7^, ^8^, ^9^ and machine learning (ML) techniques^2^ 10, 11, 12, 13, 14, 15 have been developed to predict intraoperative or surgical case duration with varying degrees of success. In previous work, we developed, tested, and validated an ML technique for predicting the remaining intraoperative duration, using preoperative and intraoperative data including medications, intraoperative events, vital signs, and exhaled anaesthetic concentrations.^11^ Models trained via this technique outperformed an established standard in a global test, and in specific clinical scenarios.

It is important to highlight that this and other ML studies predicting intraoperative duration have several limitations. First, nearly all studies have exclusively relied on retrospective data.^10^, ^11^, ^12^, ^13^, ^14^, ^15^ Although retrospective data can be masked to prevent data leaks and simulate a prospective environment, extracting and cleaning complex data from the electronic health record (EHR) in real time for suitable use in an ML model presents a number of additional challenges.^16^ For example, modest latency between electronic health record (EHR) input and ML model ingestion for key variables could degrade model performance as the underlying problem is time sensitive. Second, validation cohorts in previous studies have often been limited by (a) not being temporal holdouts, (b) having a short duration (<3 months), or (c) small size (<10 000 cases). ^2^ ^10^ 13, 14, 15 Finally, deterioration in model performance over time as a result of concept or calibration drift has been repeatedly acknowledged as a potential pitfall of using ML prediction models.^17^, ^18^, ^19^ Staff turnover (particularly trainees between academic years), addition of new surgery types, and changing hospital formularies and practices are all potential sources of performance deterioration in the perioperative setting.^20^

We developed a real-time data pipeline to extract and process preoperative and intraoperative data, and then used this pipeline to train and test the previously developed ML model for predicting intraoperative duration. Our research objectives were to (a) measure model performance in a prospective setting, (b) identify major determinants of model accuracy, and (c) measure performance deterioration over time (referred to as performance drift).

## Methods

### Study setting and data sources

Data for this study included anaesthesia records from four hospitals that were part of Washington University and BJC Healthcare (St. Louis, MO, USA). These hospitals included one academic adult hospital, one academic paediatric hospital, and two community adult hospitals. The institutional review board (IRB) of Washington University (St. Louis, MO, USA) approved this study with a waiver of consent (#201910015).

### Data pipeline

Real-time data were extracted from the Epic EHR (Verona, WI, USA) via the AlertWatch monitoring software (Ann Arbor, MI, USA). AlertWatch is a third-party clinical decision support platform^21^ for operating rooms that has previously been implemented at the study location and is currently being utilised for supporting intraoperative telemedicine.^22^

Selected fields from the AlertWatch system were transferred to a research server every minute. From the research server, data were contemporaneously cleaned and tokenised for entry into the ML model. Model predictions were made every minute during every ongoing anaesthetic. Tokenised input tensors were stored offline for calculation of performance. The data pipeline was activated M-F from 07:00 to 19:00 each day, with sporadic downtime.

### Patient selection and variable definitions

All anaesthetics captured by the data pipeline between 1 October 2022 and 30 November 2023 were included in this study. The target outcome variable was total anaesthesia time, defined as the time between anaesthesia start and anaesthesia stop time. Surgeries were scheduled with a specified case start and stop time; the difference between these (hereafter ‘scheduled duration’) was extracted as a continuous variable. Several categorical variables available before surgery were extracted for each case: procedure location, procedure urgency (elective or non-elective), surgical service, and procedure name (a descriptive text string). Of note, surgeon identity was used in previous models but was replaced with surgical service as it was not available in the real-time data pipeline.

Several intraoperative variables were extracted. Elapsed time was defined as the number of minutes between anaesthesia start and current time. Events (e.g. ‘intubation’, ‘aortic clamp applied’) were extracted with their respective time stamps. Medications were extracted with the time of administration and the action (e.g. ‘given’, ‘rate adjustment’). Six flowsheet variables were extracted: expired sevoflurane, desflurane, and nitrous oxide observations, and inspired oxygen fraction, bispectral index, and heart rate.

Latency was defined as the time difference between variable entry into the EHR and ingestion by the prediction algorithm. Latency was measured separately for event variables, medications, and flowsheet variables.

### Model performance

Cases from October 2022 to January 2023 were used to train the primary model for this study using previously described model architecture and training strategy.^11^ The ML model was a neural network. Vector embedding was used for categorical input variables, while long short-term memory (LSTM) was used for sequential inputs (e.g. flowsheet data). Embedded layers and LSTM outputs were concatenated and further treated with several dense layers. The model output was a continuous probability distribution of all possible outcomes. Further details on model architecture, including all hyperparameters, can be found in Supplementary Materials 1.

Continuous ranked probability score (CRPS) was utilised to measure model performance.^23^ CRPS is a probabilistic generalisation of absolute error that we have previously used to measure model performance in this context.^11 12^ CRPS has two important qualities that make it an effective measure of performance. First, it is measured in units of minutes and has a minimum (and optimal) score of 0. Second, it can be used to compare probabilistic predictions with deterministic predictions such as scheduled duration; for the latter, CRPS simplifies to absolute error.

The bias-corrected scheduled duration was chosen as a benchmark against which model performance was compared. First, least squares linear regression was performed on actual duration and raw scheduled duration using cases in the training dataset. This linear transformation then was applied to scheduled durations during the study period to calculate the bias-corrected scheduled duration.

Global performance of the primary model was measured and compared with bias-corrected scheduled duration from February 2023 to November 2023. Performance was also stratified by month, hospital, surgical service, scheduled duration, and percentage of actual surgery duration elapsed.

### Performance drift

Performance drift, or deterioration of performance over time, was measured in two ways. First, performance over the 10-month period was measured. Linear regression was used to quantify any decline in the ratio of bias-corrected scheduled duration CRPS and model predicted CRPS. Data were batched by week to reduce the effect of outliers.

**Fig 1:**
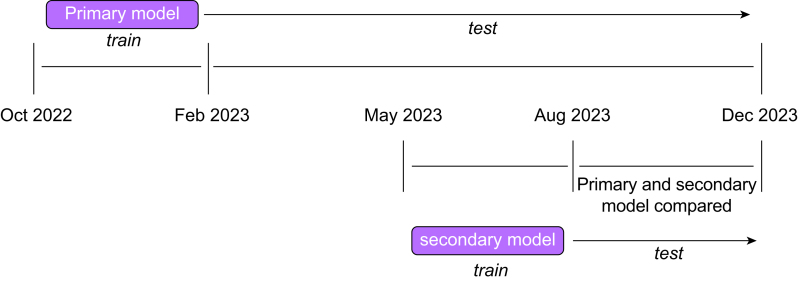
Primary and secondary model training and testing time frames.

In addition, we tested the effect of using more proximal training data by training a secondary model. Cases from May 2023 to July 2023 were used to train the secondary model, using the same model architecture, training technique, and hyperparameters as the primary model. The secondary model was not exposed to any training data before May 2023. This training window was chosen to approximate the same number of training cases as the primary model. We then compared the performance of the primary and secondary model on cases from August 2023 to November 2023, hypothesising that performance drift would be ameliorated by more proximal training data (Fig 1).

## Results

### General characteristics

Between October 2022 and November 2023, 119 983 anaesthetics were performed at the study locations, and 89 170 anaesthetic records were captured by the data pipeline (74%). Some 27 028 records were used to train the primary model, which made 6 173 435 predictions between February 2023 and October 2023 on 62 142 cases. A total of 25 558 records between April 2023 and July 2023 were used to train the secondary model. The primary and secondary models were compared for 2 621 047 predictions on 25 441 anaesthetic records between August 2023 and November 2023. Patient and procedure characteristics are summarised in Table 1.

**Table 1:** Training and testing dataset characteristics. sd, standard deviation.

	Primarymodel training	Secondarymodel training	Primary modeltest dataset	Comparativetest dataset	Cases notcaptured
Number of cases	27 028	25 586	62 142	25 689	30 813
Number of predictions	2 542 430	2 489 913	6 173 435	2 649 562	0
Median duration (min)	83	76	76	77	71
Mean duration (sd)	123.6 (sd 126.4)	116.5 (sd 117.6)	117.9 (sd 120.6)	119.2 (sd 124.1)	112.3 (sd 136.8)
Unique procedure names	20 465	19,141	42 709	19 312	Unknown
Unique anaesthetising locations	160	162	166	159	168
Facility: number of cases					
Academic adult hospital	15 359	13 467	34 774	14 289	17 069
Academic paediatric hospital	4047	4264	10 433	4090	5441
Community hospital A	5291	4513	11 688	4819	5334
Community Hospital B	1694	1581	4076	1749	2329

### Real-time data pipeline

Observed latency between EHR data entry (i.e. by the clinician) and its use of such data in making predictions was 3.22 min for medications (standard deviation [sd], 1.13 min), 3.26 min for events (sd, 1.17 min), and 2.72 min for flowsheet data (sd, 1.08 min).

### Primary model accuracy

**Fig 2:**
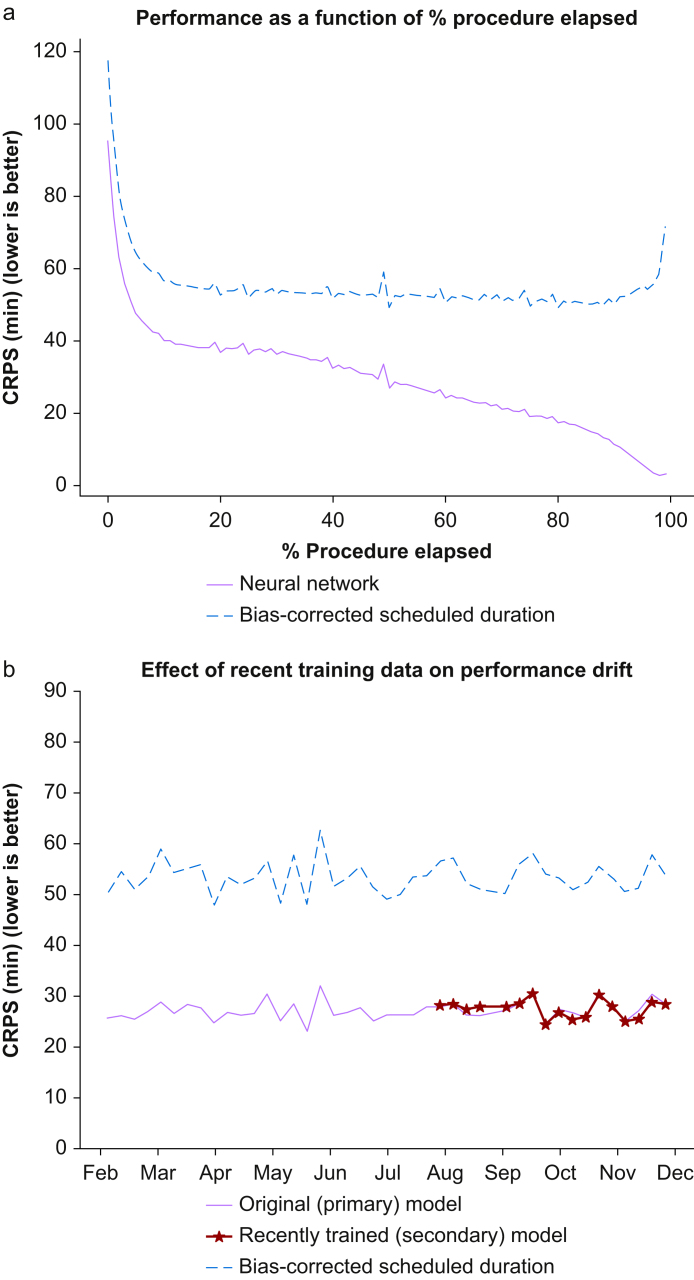
(a) Performance of primary model as a function of % procedure elapsed. At very low and high percentage of total duration, only longer cases (with higher errors) are sampled. (b) Performance of primary and secondary models over time. The primary model was tested for 10 months, including the 4-month period where primary and secondary models were compared head-to-head. CRPS, continuous ranked probability score.

**Fig 3:**
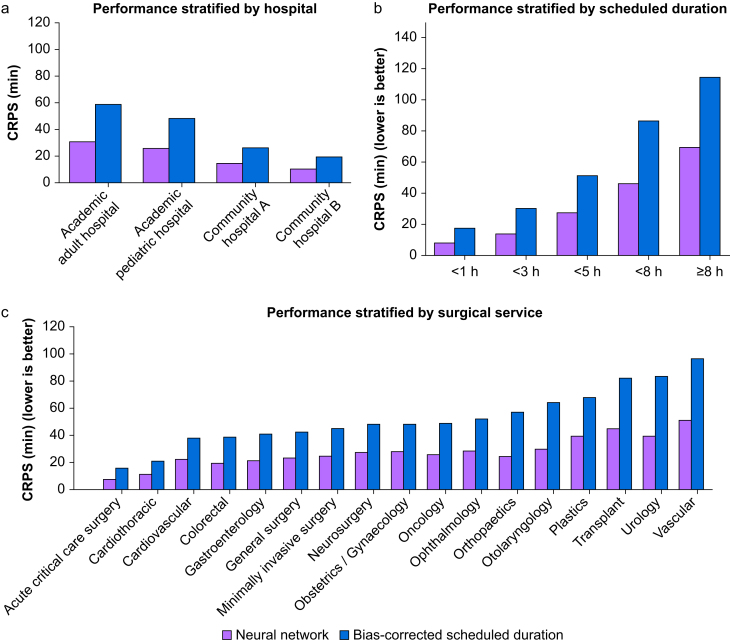
(a) Stratified performance by hospital, (b) scheduled duration, (c) surgical service (services with <100 000 predictions were excluded). CRPS, continuous ranked probability score.

Linear regression between scheduled duration and actual duration of the training dataset yielded a slope of 0.987 (standard error [se], 0.003) and intercept of −4.419 (se, 0.593). *Post hoc* sensitivity analysis using absolute value linear regression was also performed without meaningful difference (see Supplementary Materials 1). The mean CRPS across all predictions was 27.19 mins (se, 0.016 min), compared with 51.66 min (se, 0.029 min) for the bias-corrected scheduled duration. Accuracy of predictions increased as the procedure progressed towards its conclusion (Fig 2). There was modest variance in both CRPS of bias-corrected scheduled duration and model prediction, but the ratio of the two performance measures had very little variation (maximum 0.58, minimum 0.47) across weeks. Performance was also stratified by hospital, surgical service (excluding 16 out of 33 total services with <100 000 predictions), and scheduled duration (Fig 3). Both CRPS of bias-corrected scheduled duration and model prediction increased with scheduled duration. Ratio of model prediction CRPS to bias-corrected scheduled duration CRPS was between 0.4 and 0.6 for all included surgical services.

### Performance drift

The mean ratio of predictive model CRPS to bias-corrected scheduled duration CRPS was 0.24 (median, 0.54). Linear regression on this ratio demonstrated a slope of 0.0003 per week (95% confidence interval, 0.0003–0.0009). Mean CRPS for the primary model was 27.62 min (se, 0.024 min) *vs* 27.41 min (se, 0.024 min) for the secondary model. Performance over time is shown in Fig 3.

## Discussion

Based on a prospective implementation and evaluation, we found that the performance of an ML approach for predicting intraoperative duration was preserved. In this study, the ML model had a mean error of 27.19 min, approximately half the error of the bias-corrected scheduled time. Model performance was measured over a 10-month period that included the transition between academic years, a period of time associated with high staff turnover. Linear regression models showed that there was no measurable decline in model performance over this period.

Mean CRPS for both bias-corrected scheduled duration and predicted duration increased with longer scheduled duration. In addition, mean CRPS for both scheduled and predicted durations was higher overall compared with our prior study. This is likely because in the prior study, each case was weighted equally to calculate this performance metric. In a real-world setting, longer cases generate more predictions as every ongoing case generates one prediction per minute. Because longer cases have higher error, as noted above, this drives the mean error higher for both scheduled and predicted error.

The secondary model (trained with proximal data) did not yield a meaningful improvement over the primary model. This gives credence to the resilience of the model against performance drift over the measured time period. Annual retraining is likely to be sufficient in maintaining optimal performance for this type of model. The biggest advantage in use of the real-time model comes from recognition of anaesthetics that are almost completed. Patterns in the anaesthetic record that lead to case completion (such as return of spontaneous respiration, use of neuromuscular reversal agents, and administration of antiemetics) are likely to be durable over time. This likely explains our principal findings.

The data pipeline used for model implementation incorporated clinical data variables at different temporal frequencies including static preoperative variables, vitals (per minute), clinical events (every few minutes), and medications and anaesthetics (every few minutes). Traditionally, these data variables are rarely available in standard institutional pipelines (e.g. HL7) and hence implementing such ML models within EHR systems has traditionally been challenging.

This study had important limitations. First, the data pipeline is reliant on AlertWatch which is not available in the majority of perioperative EHRs. Although the reliance on the third-party infrastructure limits generalisable implementation, it also highlights the current informatics challenges of deploying ML tools at the point-of-care that utilise high-frequency, temporal data elements. We are currently exploring emerging tools for deployment of models using high throughput real-time data.^16^ Second, although the majority of cases in the four study hospitals were captured, there were resource limitations that prevented data capture of all anaesthetics. Third, our technique for capturing real-time data lacks certain data elements such as the surgeon identity and does not contain a structured vocabulary for procedure names. This limits the ability to use certain statistical techniques to predict intraoperative time (e.g. the combination of surgeon/procedure and scheduled duration), and decreases the generalisability of this technique, particularly in institutions where such data elements are readily available.

Further technical work is needed to increase the breadth of data capture and reduce downtime. As with prior studies, prediction of turnover time and prediction of the duration of sequences of cases are outside the scope of this algorithm. Real-world deployment of an intraoperative duration prediction model would require accompanying perioperative process changes, such as modification to allocated surgeon time, to fully utilise its benefits.^24^

In conclusion, these findings demonstrate that continuous prediction of intraoperative duration using a neural network is preserved in a real-world setting. Furthermore, performance across a prolonged test period is resistant to performance drift. Further investigation is required to determine the potential value of such an algorithm in improving perioperative outcomes.

## Authors’ contributions

Study conceptualisation: YJ, TK.

Model/data pipeline design: YJ.

Model/data pipeline implementation: YJ.

Model/data pipeline testing: YJ.

Critical discussion of results: YJ, TK.

Drafting of manuscript: YJ.

Review of manuscript: TK.

## Declarations of interest

The authors declare that they have no conflicts of interest.
