# Supplementary material for: Implementation and prospective performance evaluation of an intraoperative duration prediction model using high throughput real-time data
Source: BJA Open. 2024 May 7;10:100285. doi: 10.1016/j.bjao.2024.100285 (PMC11091514; doi:10.1016/j.bjao.2024.100285)
Supplement: Multimedia component 1 [file mmc1.docx]

**Supplemental Materials 1**

**A. Model Details**

**Software:** All model testing, and calculation of performance was done using Python 3.10.4 and the accompanying packages tensorflow 2.11.0, tensorflow probability 0.19.0, numpy 1.24.1, and scipy 1.10.0.

**Machine Learning Model Architecture:**


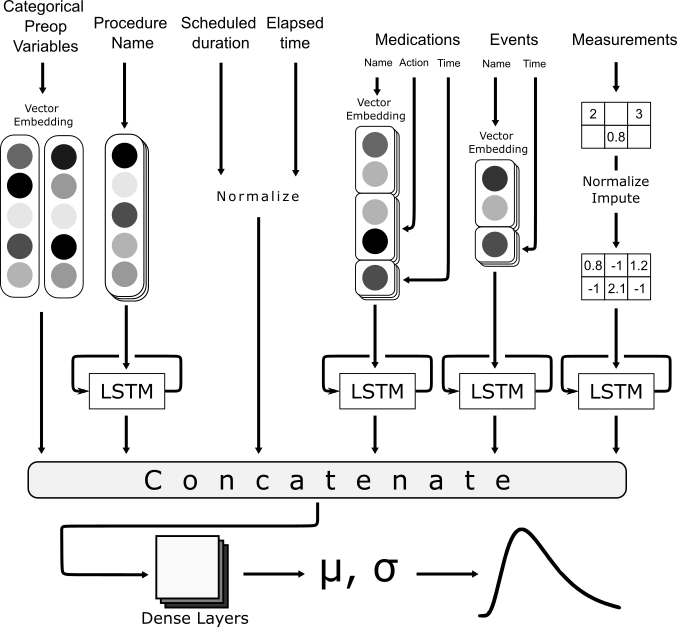


**Hyperparameters:**

Boolean variables (i.e. emergent / non-emergent status) were carried over unmodified into the main concatenation layer.

Continuous input variables (scheduled duration, elapsed time) were normalized to have mean 0 and standard deviation 1, and carried into the main concatenation layer.

Categorical inputs variables were treated using vector embedding. The hyperparameters are as following:

| Categorical Variable | Vocabulary Size | Embedding Dimension |
| --- | --- | --- |
| Location | 250 | 4 |
| Surgical Service | 30 | 4 |
| Anesthesia Provider* | 200 | 4 |

Sequential inputs were treated with LSTM. The hyperparameters are as following:

| Sequential Variable | Vocabulary Size | Word Embedding Dimension | Maximum Sequence Length | LSTM Output Dimension |
| --- | --- | --- | --- | --- |
| Events^†^ | 100 | 16 | 15 | 16 |
| Medications^†^ | 250 | 12 | 25 | 16 |
| Medication Action^**^ | 5 | 4 | 25 | N/A |
| Procedure Name | 700 | 8 | 15 | 16 |
| Flowsheet Measurements | *** | N/A | 45 | 16 |

All outputs from categorical variable embedding and sequential input LSTMs were carried into the main concatenation layer.

**Additional Details:**

^*^ Anesthesia provider refers to any anesthesia provider logged into the case in the EHR, at the time of the prediction. For instances where multiple anesthesia providers were logged into the case (i.e. attending / CRNA or attending / trainee), the mean of the embedded outputs were carried into the concatenation layer.

^**^ Medication Action refers to ‘given’, ‘rate modified’, etc. This variable accompanies the medication name. The embedded outputs were concatenated with the medication name embedded output before treatment by LSTM.

^***^ Flowsheet measurements is a composite input of 6 continuous flowsheet variables: heart rate, FiO2, BIS Monitor, expired NO2, expired sevoflurane, and expired desflurane. The value of each of these variables was encapsulated every minute into a length-6 vector normalized to mean 0, standard deviation 1, and missing values as -1. Thus, with maximum sequence length 45, the model is at any point reading 45 minutes of the most recent flowsheet data.

^†^ Event and medications names were first embedded, then concatenated with the time of the elapsed time of their occurrence before treatment by LSTM.

The main concatenation layer was used as input for 3 dense layers (dimensions 64, 32, and 16 with rectified linear unit activation function), feeding into the final output layer. The final output layer had two nodes, corresponding to the mean and standard deviation of a lognormal distribution. The loss function was the negative log likelihood of the label (i.e. actual anesthesia time) predicted by the output lognormal distribution.

**Training:**

The Adam optimizer was used for training, with learning rate set to 0.0001. Training cases were randomized and batched into groups of 32. The model was trained for 200,000 batches.

**B. Bias Correction of Scheduled Duration**

Mean CRPS of the raw scheduled duration was 51.86 minutes. Bias correction was performed using least squares linear regression which yielded a slope of 0.987 and intercept of -4.419. Mean CRPS of this bias-corrected scheduled duration was 51.66 minutes. Post-hoc sensitivity analysis was performed using absolute value linear regression, which yielded a slope of 0.93 and intercept of -2.9. Mean CRPS of the alternate bias-corrected scheduled duration was 52.99 minutes.

The scipy python package was used to perform these calculations.
